# Two Variants in *SLC24A5* Are Associated with “Tiger-Eye” Iris Pigmentation in Puerto Rican Paso Fino Horses

**DOI:** 10.1534/g3.117.043786

**Published:** 2017-06-27

**Authors:** Maura Mack, Elizabeth Kowalski, Robert Grahn, Dineli Bras, Maria Cecilia T. Penedo, Rebecca Bellone

**Affiliations:** * Veterinary Genetics Laboratory, School of Veterinary Medicine, University of California, Davis, California 95616; † Department of Biology, University of Tampa, Florida 33606; ‡ Centro de Especialistas Veterinarios, San Juan, Puerto Rico 00926; § Population Health and Reproduction, School of Veterinary Medicine, University of California, Davis, California 95616

**Keywords:** *Equus caballus*, iris, Paso Fino horse, pigmentation, SLC24A5

## Abstract

A unique eye color, called tiger-eye, segregates in the Puerto Rican Paso Fino (PRPF) horse breed and is characterized by a bright yellow, amber, or orange iris. Pedigree analysis identified a simple autosomal recessive mode of inheritance for this trait. A genome-wide association study (GWAS) with 24 individuals identified a locus on ECA 1 reaching genome-wide significance (*P*_corrected_ = 1.32 × 10^−5^). This ECA1 locus harbors the candidate gene, *Solute Carrier Family 24* (*Sodium/Potassium/Calcium Exchanger*), *Member 5* (*SLC24A5*), with known roles in pigmentation in humans, mice, and zebrafish. Humans with compound heterozygous mutations in *SLC24A5* have oculocutaneous albinism (OCA) type 6 (OCA6), which is characterized by dilute skin, hair, and eye pigmentation, as well as ocular anomalies. Twenty tiger-eye horses were homozygous for a nonsynonymous mutation in exon 2 (p.Phe91Tyr) of *SLC24A5* (called here Tiger-eye 1), which is predicted to be deleterious to protein function. Additionally, eight of the remaining 12 tiger-eye horses heterozygous for the p.Phe91Tyr variant were also heterozygous for a 628 bp deletion encompassing all of exon 7 of *SLC24A5* (c.875-340_1081+82del), which we will call here the Tiger-eye 2 allele. None of the 122 brown-eyed horses were homozygous for either tiger-eye-associated allele or were compound heterozygotes. Further, neither variant was detected in 196 horses from four related breeds not known to have the tiger-eye phenotype. Here, we propose that two mutations in *SLC24A5* affect iris pigmentation in tiger-eye PRPF horses. Further, unlike OCA6 in humans, the Tiger-eye 1 mutation in its homozygous state or as a compound heterozygote (Tiger-eye 1/Tiger-eye 2) does not appear to cause ocular anomalies or a change in coat color in the PRPF horse.

In mammals, eye color is determined by the quantity and packaging of melanin in the iris. The iris is comprised of connective tissue and pigmented cells that are involved in controlling the amount of light entering through the eyeball, reaching the retina, and thus enabling vision. Human iris color variation research has determined that melanocyte abundance is similar between brown and blue irides (Imesch *et al.* 1997); however, blue irides were reported to have less eumelanin and pheomelanin than darker eye colors (Prota *et al.* 1998; Wielgus and Sarna 2005). This suggests that genes involved in the production of melanin are likely the best candidates for iris color variation.

The genetics of iris color in humans has been historically investigated as a simple Mendelian trait, with blue eyes explained as a recessive trait. However, this hypothesis fails to explain intermediate colors such as green and hazel, and does not explain the reported instances in which blue eyes do not follow a simple recessive Mendelian inheritance pattern (Sturm and Larsson 2009). That being said, the majority of human blue/brown iris variation has been explained by mutations in two loci, *melanosomal transmembrane protein* (*OCA2*) and the linked gene on HSA 15, *HECT and RLD domain containing E3 ubiquitin protein ligase 2* (*HERC2*) (Liu *et al.* 2009). In addition, several other genes have been associated with brown/blue human iris color variation including *MC1R*, *TYR*, *SLC24A4*, *KITLG*, *TYRP1*, and *IRF4* (Gudbjartsson *et al.* 2008; Han *et al.* 2008; Kayser *et al.* 2008; Sulem *et al.* 2007, 2008).

Dilution of iris pigmentation is often associated with ocular anomalies. Oculocutaneous albinism (OCA), a group of genetic disorders in humans, is characterized by hypopigmentation of the hair, skin, and iris. Patients with OCA have also been diagnosed with decreased visual acuity, photophobia, color vision impairment, and strabismus. There are seven types of OCA (OCA1–7) resulting from known mutations in different genes affecting pigmentation (King *et al.* 2003; Lee *et al.* 1994; Manga *et al.* 1997; Newton *et al.* 2001; Kausar *et al.* 2013; Gronskov *et al.* 2013; Wei *et al.* 2013). Understanding the mechanism for the variation in iris pigmentation and the potential effect on vision has important implications across mammalian species.

In the horse, some knowledge on iris color variation has come from studying coat color loci. While the eye color of most horses has been described as black or dark brown, horses with blue and amber eyes have also been described. Horses homozygous for the cream dilution allele, caused by a missense mutation in *solute carrier family 45 member 2* (*SLC45A2*), have blue irises (Mariat *et al.* 2003). Also, several white coat pattern mutations (known as tobiano, overo, and splashed white) (Brooks *et al.* 2010; Hauswirth *et al.* 2012; Santschi *et al.* 1998) have been implicated in blue iris pigmentation. A lighter iris pigmentation phenotype, described as amber or hazel, has also been documented for some horses heterozygous for the cream dilution (Sponenberg 2009). In addition, all horses homozygous and heterozygous for the champagne dilution allele, a missense mutation in *solute carrier* 36 *family 36 member A1* (*SLC36A1*), have a dilute coat color and amber eyes (Cook *et al.* 2008). Beyond the listed coat color-specific iris variants, no other studies have specifically investigated the genetics of iris pigmentation variation in horses.

Ocular anomalies have been documented to correlate with pigmentation traits in the horse. For example, congenital stationary night blindness is associated with leopard complex spotting (Sandmeyer *et al.* 2012; Bellone *et al.* 2013), and multiple congenital ocular anomalies are associated with the silver dapple coat color dilution (Grahn *et al.* 2008; Andersson *et al.* 2013). There have been no studies that investigate ocular anomalies associated with iris color variation when not accompanied by a coat color dilution.

Here, we report the first study to unravel the genetics of an iris color variant with no known coat dilution or white spotting in horses. A unique iris color, called tiger-eye (also referred to by some breeders as “goat-eye”), segregates in the PRPF horse breed. The PRPF is an isolated island population believed to have originated from the importation of Spanish horse breeds and the now extinct Spanish-Jennets, and are closely related to other breeds of Spanish origin (Petersen *et al.* 2013). Tiger-eye is characterized as a yellow, amber, or bright orange-colored iris. While the PRPF breed is perhaps best known by horse enthusiasts for its distinct lateral four beat “fino” gait, tiger-eyed horses are especially valued for their striking appearance (Figure 1). Preliminary pedigree analysis suggested a simple recessive mode of inheritance. Initial candidate gene investigation of the human iris color contributors, *HERC2* and *OCA2*, suggested promising results for association with *HERC2* (*P* = 0.02). However, these were not replicated upon testing additional horses (Kowalski and Bellone 2011). Here, we expand our initial pedigree analysis and perform a GWAS to identify other candidate loci for investigation.

**Figure 1:**
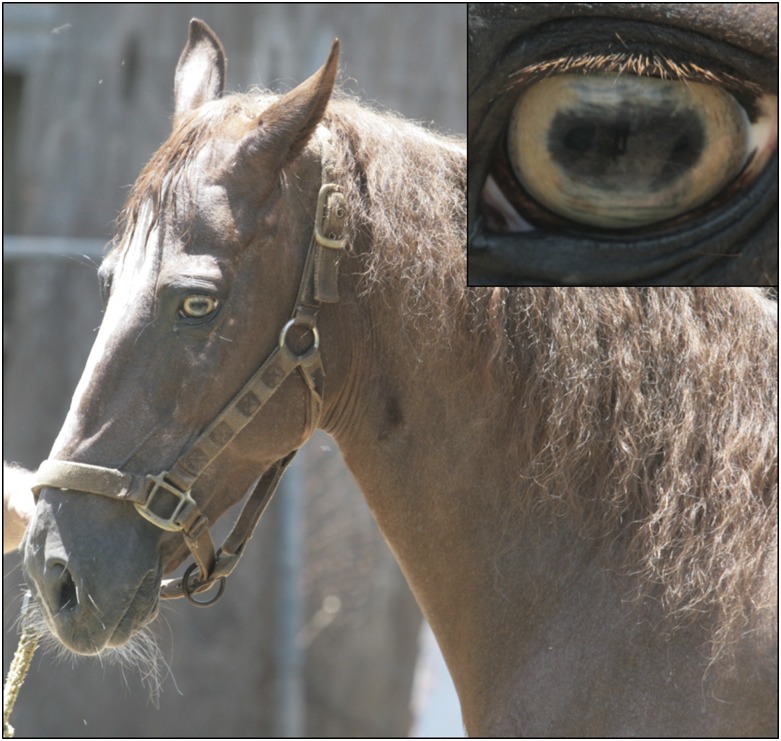
Tiger-eye iris phenotype in the PRPF horse. Tiger-eye is defined as a lighter iris shade characterized by bright orange, amber, or yellow as shown here.

## Materials and Methods

### Horses and phenotyping

**Figure 2:**
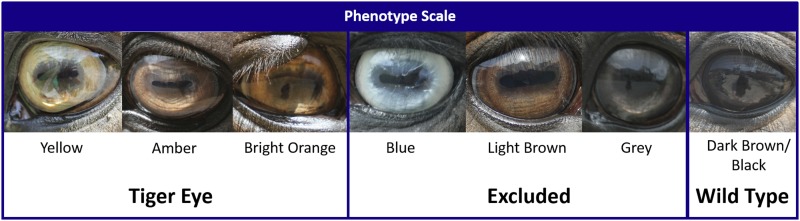
Phenotype scale utilized for iris color variation. Yellow, amber, and bright orange irides were considered to be tiger-eyed. Horses with dark brown/black eyes were considered to be the wild-type phenotype. Blue, light brown, and gray were excluded from this study.

Bilateral photographs of eyes were collected from 216 available PRPF horses. Additional information was collected for each horse enrolled in the study, and included four-generation pedigree information, coat color, date of birth, and sex. Owner-reported coat color was confirmed by visual analysis of photographs provided. Bilateral photographs of each horse were visually examined and eye-color phenotypes were independently assigned by two authors (E. K. and M. M.) based on color and shade as follows: blue, yellow, amber, bright orange, light brown, gray, or dark brown/black (Figure 2). Horses were included in the study if the assigned phenotype was confirmed by both observers.

Yellow, amber, and orange-eyed horses were considered to have the tiger-eye phenotype and the final sample set included 32 tiger-eye samples. Light brown or gray eye colors were excluded from the study because they were intermediary colors that could not be easily categorized as darker shades of the tiger-eye phenotype or lighter shades of the wild-type phenotype. Since blue eyes in horses have been associated with white spotting patterns and homozygosity for the cream dilution, these were also initially excluded from analysis (Mariat *et al.* 2003; Hauswirth *et al.* 2012). Additionally, because base coat color dilution genes affect iris pigmentation in horses, horses with a dilute coat color phenotype were also excluded from the study (Mariat *et al.* 2003). Sixty-two horses were excluded based on these criteria. After exclusions, 122 PRPF horses had the dark brown/black eye color, which is considered wild-type. Total genomic DNA for array genotyping and Sanger sequencing from the owner-supplied hair samples was extracted using a modified protocol for the Gentra Puregene DNA Isolation Kit (QIAGEN, Valencia, CA). Specifically, 15–20 hair bulbs were placed in 300 µl of Puregene Cell Lysis Solution containing 0.003 mg of proteinase K (Sigma-Aldrich) and incubated at 55° overnight. The sample was then cooled to room temperature and the remainder of the manufacturer’s protocol was followed using 99 µl of Protein Precipitation Solution and precipitating the DNA in 300 µl of isopropanol. Precipitated DNA was resuspended in 60 µl of DNA Hydration Solution. DNA utilized only for genotyping the identified variants was isolated according to the crude hair lysis protocol described in Locke *et al.* (2002). DNA was extracted from a total of 173 (tiger-eye horses = 32, wild-type horses = 122, excluded blue-eyed horses = 9, and excluded dilute coat color horses = 10) PRPF horses and 196 horses from four other breeds.

### Pedigree analysis

Pedigree data were either provided by owners or determined from an online PRPF pedigree database (Crossley 1998). Pedigrees were evaluated and visualized using the program Pedigraph (Garbe and Da 2008) to identify common ancestors among all affected individuals, and investigate multiple modes of inheritance. To further test an autosomal recessive inheritance, individual pedigrees were extracted for each of the 32 confirmed tiger-eyed individuals, looking for common ancestors that could be found on each side of the pedigree.

### GWAS

A GWAS with 24 horses (14 tiger-eye and 10 wild-type) was performed using the Illumina Equine SNP70 BeadChip. Genotyping was performed by GeneSeek (Lincoln, NE). Analysis and visualization of data were performed using Golden Helix SNP & Variation Suite v8 (Golden Helix, Bozeman, MT). After quality control filtering (sample call rate > 0.95, minor allele frequency > 0.05, and SNP call rate > 0.90), 41,820 informative markers remained. To minimize confounding results due to relatedness and maximize power with a small sample set, samples were selected for GWAS based on one degree of separation and data were initially analyzed by a χ^2^ test under a recessive model. However, in our initial analysis our genomic inflation was significant (λ = 1.21), indicating that the sample selection criteria lacked sufficient stringency to minimize the impact of relatedness in this island population. Genome-wide identity by descent (IBD) was calculated to visualize the relatedness in the population (Supplemental Material, Figure S1 in File S1). To address the unequal relatedness in the case and control groups, a single locus mixed linear model (SLMM) approach was utilized (Golden Helix). The SLMM utilizes an identity by state (IBS) to estimate population structure and Efficient Mixed-Model Association eXpedited (EMMAX) is utilized to calculate *P*-values using an *F* test for a recessive model (Kang *et al.* 2010). Loci reaching a strict Bonferroni correction for multiple testing were considered further (*P* < 1.16 × 10^−6^).

### Replication testing

To confirm the initial association identified on ECA1 by the GWAS, four markers (BIEC2_60719, BIEC2_61330, UKUL310, and BIEC2-61972) from this locus were tested in 22 additional horses. The replication sample set was comprised of 11 tiger-eye individuals and 11 brown-eyed individuals. These additional horses were selected based on one degree of separation. SNPs were genotyped by PCR-RFLP testing. Primers and restriction enzymes can be found in Table S1 in File S1. First, 10 μl of PCR products were digested for 6 hr at the manufacturer recommended temperature for each enzyme utilized. Products were then resolved on 1.5% agarose gels stained with ethidium bromide (EtBr) or fluorescently labeled and visualized on an ABI 3730 Genetic Analyzer (Grand Island, NY). To test for association with the tiger-eye phenotype, χ^2^ tests were performed using a recessive model.

### Candidate genes

The 3.5 Mb region of association on ECA 1 includes two candidate genes with known roles in pigmentation, namely *SLC24A5* and *myosin VA* (*MYO5A*). Mutations in MYO5A have been shown to cause Griscelli syndrome in humans and Lavender foal syndrome in horses (Menasche *et al.* 2003; Brooks *et al.* 2010). In Lavender foal syndrome, a single nucleotide deletion in exon 30 causes a dilute coat color and fatal neurological deficits (Brooks *et al.* 2010). Griscelli syndrome is most frequently associated with dilute pigmentation as well as immunologic or neurological symptoms; however, one patient homozygous for a *MYO5A* F-exon deletion exhibited hypopigmentation without the immune and neurological deficits (Menasche *et al.* 2003). Mutations in MYO5A are frequently associated with immune or neurological deficits therefore *MYO5A* was not investigated further.

*SLC24A5* is also located in the region of interest. Polymorphisms in this gene have been shown cause a golden phenotype in zebrafish and have been associated with variations in skin color (Lamason *et al.* 2005), as well as OCA6, in humans (Stokowski *et al.* 2007; Morice-Picard *et al.* 2014). Additionally, *SLC24A5* causes a novel form of ocular albinism in mice (Vogel *et al.* 2008). Given the known roles of *SLC24A5* in pigmentation and specifically ocular pigmentation, we chose to investigate this gene further.

### Candidate gene sequencing and investigation

Sanger sequencing of two tiger-eye and one wild-type horse was performed for all nine predicted exons and flanking introns of *SLC24A5*, a functional candidate gene located in the associated region on ECA1. Predicted exon and intron structure was determined from the Ensembl database entry (transcript ID: ENSECAT00000019866.1). Primer sequences can be found in Table S1 in File S1. The PCR protocol was performed with a total volume of 10 µl using 4.0 pmol of primers, 25 ng of DNA, 1× PCR buffer with 2.0 mM MgCl2, 100 µM of each dNTP, and 0.1 U FastStart Taq DNA polymerase (Roche Applied Science Indianapolis, IN). PCR products were visualized on a 1% EtBr agarose gel to verify correct product size before sequencing. The amplicon was gel purified using a QIAquick Gel Extraction Kit following the manufacturer’s recommendations (QIAGEN). Amplicons were subsequently sequenced using BigDye Terminator v1.1 and products detected using an ABI 310 Genetic Analyzer (Applied Biosystems, at ThermoFisher Scientific, Grand Island, NY). To identify variants among the samples sequenced, data were analyzed using Sequencher version 5.2.4 (Gene Codes, Ann Arbor, MI). EquCab 2 assembly was used as the reference sequence.

Genotyping for the exon 2 missense mutation (A > T, p.Phe91Tyr) and exon 7 deletion were performed by allele-specific PCR using fluorescent labeled primers. PCR was performed in a total volume of 10 µl using 2.0 pmol of primers, 25 ng of DNA, 1× PCR buffer with 2.0 mM MgCl^2^, 100 µM of each dNTP, and 0.1 U FastStart Taq DNA polymerase (Roche Applied Science). Amplicons were visualized on an ABI 3730 Genetic Analyzer (Applied Biosystems, at ThermoFisher Scientific). Initially, 154 PRPF horses (32 tiger-eye and 122 wild-type) were genotyped for both mutations. In an effort to detect a homozygote for the exon 7 mutation, an additional set of 19 PRPF horses that were previously excluded due to blue eye color or coat color dilution were genotyped for both mutations. In total, 196 horses from four additional breeds (Colombian Paso *N* = 90, Mangalara *N* = 20, Lusitano = 44, and Andalusian *N* = 42) of unknown eye-color phenotypes were genotyped for both polymorphisms. These breeds were selected based on a close phylogenetic relationship to PRPF, as reported by Petersen *et al.* (2013). To determine if the Phe > Tyr substitution is deleterious, the known and predicted amino acid sequence for this protein was compared across 100 vertebrates using the USCS genome browser (https://genome.ucsc.edu/). The consensus classifier Predict SNP was utilized to computationally determine if this was a neutral or deleterious substitution (Bendl *et al.* 2014). Variants were named according to Ensembl transcript and UniProt accession numbers (ENSECAT00000019866.1 and F7BP61).

### Ophthalmic examination

A board-certified veterinary ophthalmologist (DB) examined three tiger-eye and three wild-type PRPF horses. A complete ophthalmic examination was performed including menace response, dazzle response, pupillary light reflexes, applanation tonometry (Tonopen XL; Mentor, Norwell, MA), biomicroscopy (SL-15 Portable Slit Lamp, Kowa, Japan), indirect ophthalmoscopy, and cranial nerve examination. Careful evaluation of possible pigment variations of the ocular structures was performed.

### Data availability

GWAS data from this study including map and ped files were uploaded to Figshare and are available at https://doi.org/10.6084/m9.figshare.5146090.v1. Variants detected in *SLC24A5* can be found in Table 3.

## Results

### Pedigree and coat color analysis

Of the 216 PRPF horses sampled, parental iris color phenotype could be confirmed for 41 horses (dark brown/black = 29, tiger-eye = 12). These were used to examine modes of inheritance for this trait. In five matings of tiger-eye crossed with tiger-eye, all offspring had the tiger-eye phenotype (Table S2 in File S1 and Table 1). The other mating combinations resulted in tiger-eye or wild-type offspring in the ratios expected by a simple recessive trait (Table S2 in File S1 and Table 1). These data are consistent with brown iris color being dominant to tiger-eye. All three base coat colors described in horse are represented in the tiger-eye population data set and no obvious trend between shade of tiger-eye color (yellow *vs.* amber *vs.* orange) and coat color was observed (Table S3 in File S1). Tiger-eye was detected in more females (*N* = 23), than males (*N* = 9) in our sample set; however, this ratio is representative of the ratio of males and females sampled in our total population (female = 94 and male = 60).

**Table 1:** Pedigree analysis for tiger-eye phenotype

Mating Type	Brown Progeny	Tiger Progeny
Tiger × tiger	0	5
Tiger × brown	18	6
Brown × brown	11	1

Further support for a recessive mode of inheritance was identified by examining the most recent common ancestor of all tiger-eye horses. Sire X386 could be found on both the maternal and paternal sides of the pedigree for 31 out of the 32 confirmed tiger-eyed individuals within 10 generations. For individual 09–97, sire X386 was present on the paternal side, but based on the reported pedigree information could not be identified on the maternal side of the pedigree.

### GWAS

**Figure 3:**
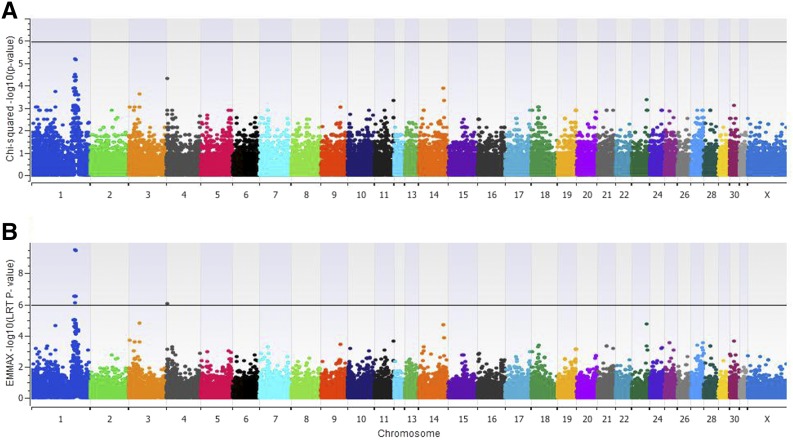
Manhattan plots. (A) Manhattan plot of χ^2^ association test using a recessive model. (B) Manhattan plot of single locus mixed linear model analysis for tiger-eye phenotype in horses. Shown are the output –log_10_ *P*-values calculated from the linear regression and *F*-test. The black line represents the threshold for strict Bonferroni level of significance (*P* < 1.16 × 10^−6^). EMMAX, Efficient Mixed-Model Association eXpedited.

Utilizing a recessive model and a small sample set (*N* = 24), a locus on ECA1 was strongly associated with the tiger-eye phenotype (*P* = 6.76 × 10^−6^, Figure 3A and Table S4 in File S1). However, under this analysis, none of the SNPs from this locus reached genome-wide significance (*P* < 1.26 × 10^−6^). In addition, significant genomic inflation was detected with λ = 1.21 (Figure S1 in File S1). To visualize the unequal genetic relatedness in our population giving rise to the inflation, a genome-wide IBD analysis was performed, which revealed that the brown-eyed horses in our population were more closely related to each other than the tiger-eye horses (Figure S1 in File S1).

To correct for the genomic inflation, we implemented a SLMM, specifically EMMAX. This model uses IBS to represent population structure and performs a linear regression to correct for the substructure. Under this model, seven SNPs on ECA1 reached genome-wide significance after correction for multiple testing (*P*_corrected_ = 1.32 × 10^−5^, Figure 3B). This locus spans 3.5 Mb and contains over 41 genes, two of which are functional candidates.

To further investigate this locus, an additional 22 horses were tested for four of the seven most significantly associated SNPs (BIEC2_61330, UKUL310, BIEC2_60719, and BIEC2_61972) by PCR-RFLP. These SNPs were chosen because of their location in relation to the two functional candidate genes as well as the ease at which the PCR-RFLP testing could be designed and performed. The association at this locus was confirmed in this additional sample set (*P* = 7.77 × 10^−10^, Table 2).

**Table 2:** Replication testing for the tiger-eye associated markers on ECA1

Marker	Location	χ^2^ *P* Replication Set (*N* = 22)	χ^2^ *P* Combined (*N* = 46)
BIEC2_60719	chr1.138425009	7.69 × 10^−5^	7.77 × 10^−10^
BIEC2_61330	chr1.139309629	3.97 × 10^−3^	3.58 × 10^−8^
UKUL310	chr1.141657822	3.97 × 10^−3^	3.58 × 10^−8^
BIEC2_61972	chr1.141804174	0.025	6.63 × 10^−7^

chr, chromosome.

### Mutational discovery and association testing

Sanger Sequencing of two tiger-eye horses and one brown-eyed horse revealed eight polymorphisms (Table 3). Two of these variants were located in the coding sequence. A missense mutation in exon 2 (A > T, F91Y) was identified in both tiger-eye horses, and a deletion of 628 bp encompassing all of exon 7 and flanking sequences of the neighboring introns was identified in one tiger-eye horse. Using the consensus classifier PredictSNP, the nonsynonymous mutation in exon 2 (p.Phe91Tyr) (which here we will call Tiger-eye 1 allele) was predicted to be deleterious to protein function with 87% accuracy (Bendl *et al.* 2014). The 628 bp deletion encompasses all of exon 7 (named Tiger-eye 2 allele) and some of the flanking intron sequence, thus deletion of this region is predicted to cause exon 6 to be spliced to exon 8, leaving the reading frame unaltered but removing 69 amino acids.

**Table 3:** List of variants detected in *SLC24A5*

Location	Reference Allele	Alternate Allele	Location in Gene
chr1:141678582	C	T	5′ upstream
chr1:141677402	A	T	Exon 2
chr1:141665142	C	A	Intron 4
chr1:141662647	A	G	Intron 7
chr1:141660611–141661239	—	DEL	Exon 7
chr1:141659206	T	G	Intron 8
chr1:141657748	T	C	3′ UTR
chr1:141657822	A	G	3′ UTR

chr, chromosome; DEL, deletion; UTR, untranslated region.

All 154 horses (32 tiger-eye and 122 wild-type) were genotyped for both variants. The Tiger-eye 1 allele had a strong association with the tiger-eye phenotype (*P* = 7.88 × 10^−21^, Table 4). Twenty out of 32 tiger-eye horses could be explained by homozygosity for this variant (Table 4). However, the 12 remaining tiger-eye horses were heterozygous for the exon 2 allele. Eight of these were explained by compound heterozygosity (Tiger-eye 1/Tiger-eye 2) and the remaining four are left unexplained (Figure S2 in File S1). There was no visible difference in shade of eye color between Tiger-eye 1 homozygotes and the compound heterozygotes (Table S5 in File S1). None of the wild-type horses (*N* = 122) were homozygous for either allele or compound heterozygotes (Table 4). Three of these unexplained tiger-eyed horses phenotyped as orange-tiger, a shade on the darker end of the tiger-eye phenotype spectrum (Figure S2, A–C and Table S5 in File S1). All three of these outlier horses have a bay coat color and are heterozygous at the Tiger-eye 1 locus. One additional outlier was phenotyped as yellow-tiger (Figure S2D in File S1), the lightest end of the tiger-eye phenotype. This horse is now deceased and thus unavailable for visual confirmation of eye color and sample concordance.

**Table 4:** Genotyping results for the exon 2 mutation A > T and exon 7 deletion for the initial test population

	Genotype									
		*A/A −/−*	*A/A −/DEL*	*A/A DEL/DEL*	*A/T −/−*	*A/T −/DEL*	*A/T DEL/DEL*	*T/T −/−*	*T/T −/DEL*	*T/T DEL/DEL*
PRPF tiger		0	0	0	4	8	0	20	0	0
PRPF wild-type		57	9	0	65	0	0	0	0	0
Colombian Paso		90	0	0	0	0	0	0	0	0
Mangalara		20	0	0	0	0	0	0	0	0
Lusitano		44	0	0	0	0	0	0	0	0
Andalusian		42	0	0	0	0	0	0	0	0

Genotype combinations are presented with exon 2 data first (*A/A*, *A/T or T/T*) followed by exon 7 data (*−/−*, *−/DEL*, and *DEL/DEL*). DEL, deletion; PRPF, Puerto Rican Paso Fino.

**Figure 4:**
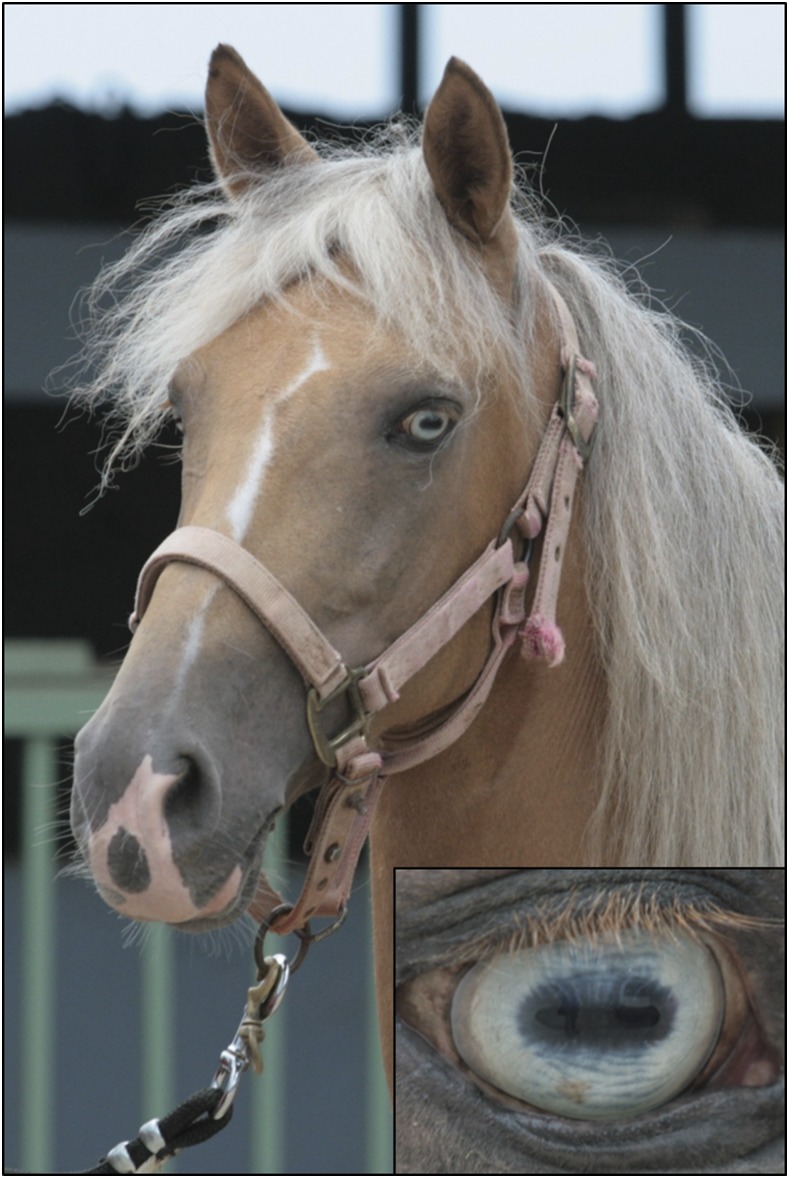
Homozygosity for *SLC24A5* exon 7 deletion. Homozygosity for the 628 bp deletion of *SLC24A5* is not lethal as evidenced by a single blue-eyed horse. The bilateral blue eye color is shown here (right eye is shown in inset).

In our initial genotyping for the Tiger-eye 2 allele, we did not detect any horses homozygous for this deletion (*N* = 154). To determine if this variant was homozygous lethal, 19 additional horses were genotyped for this polymorphism. Nine were blue-eyed and 10 had a dilute coat color and had been previously excluded from our investigation. One Tiger-eye 2 allele homozygote was detected, providing evidence that this mutation is not homozygous lethal. This horse had a dilute coat color consistent with heterozygosity for the cream mutation (known as palomino, Mariat *et al.* 2003) and had blue eyes (Figure 4). Blue eyes are characteristic of horses homozygous for the cream allele (*SLC45A2 G > A*) but not typical of heterozygotes. Thus, this horse was also tested for all known coat color variants routinely performed at the Veterinary Genetics Laboratory (UC-Davis) to determine if another variant may explain the blue eyes. Testing known loci confirmed the palomino genotype, homozygous recessive at the extension locus (*e/e*) and heterozygous at the cream locus (*Cr/N*), confirming its coat appearance, but no other variants associated with blue eyes were detected. The presence of the cream dilution may have obscured any effect that the Tiger-eye 2 variant would have in its homozygous state, therefore we cannot make any observations about the Tiger-eye 2 variant’s effect on coat color.

Tiger-eye has not been documented in any other breed other than the PRPF to date. Four breeds closely related to PRPF as defined by genetic diversity among horse breeds (Petersen *et al.* 2013) were screened for the two identified PRPF tiger-eye variants. A total of 196 horses with unexamined but presumed nontiger-eye color (90 Colombian Paso horses, 20 Mangalara horses, 44 Lusitano horse, and 42 Andalusian horses) were genotyped for the Tiger-eye 1 and 2 alleles and all were found to be homozygous for the reference alleles at both loci. This provides further support to the notion that these are causal variants for the tiger-eye phenotype in PRPF horses.

### Ophthalmic examination

Aside from the observable difference in iris pigmentation, no ocular abnormalities were detected when comparing three tiger-eyed horses (two homozygous for the Tiger-eye 1 allele and one compound heterozygote) to three wild-type controls. No visual deficits were noted and no signs of reduced pigmentation in the retina or on the retinal pigment epithelium (RPE) were observed. An eyelid margin defect and a dermoid on the dorsal limbus was observed OD in one tiger-eye horse.

## Discussion

In our population of PRPF horses, we identified two mutations in *SLC24A5* that explain 88% of the tiger-eye cases. Twenty of the tiger-eye horses were homozygous for the Tiger-eye 1 allele (c.271A > T) while eight were compound heterozygotes for Tiger-eye 1 and Tiger-eye 2 alleles (c.875-340_1081 + 82del). Of the four horses that could not be explained by these mutations, three phenotyped as Orange-Tiger, a shade on the darker end of the tiger phenotype spectrum. These may represent a gradation in the phenotype that could not be resolved and may actually be examples of a lighter shade of brown irides (Figure S2 in File S1). The one tiger-eyed horse with the yellow-tiger phenotype, was heterozygous for the Tiger-eye 1 allele and her iris color may be explained as a compound heterozygote for a yet unidentified variant in this gene. In addition, the horse is deceased, so was unavailable for resampling or additional visual inspection.

In the initial GWAS, two out of the 14 tiger-eye horses were compound heterozygotes (Tiger-eye 1/Tiger-eye1 = 12 and Tiger-eye 1/Tiger-eye 2 = 2). It is likely that we would not have successfully mapped the locus on ECA1 if there had been more compound heterozygotes in our GWAS population. These two horses could also partially explain why the *P*-values for the χ^2^ test using a recessive model did not reach the Bonferroni threshold of significance, as this method does not account for multiple alleles.

*SLC24A5* is located on chr1:141,657,837–141,678,329 and encodes, a potassium-dependent sodium–calcium ion exchanger that is believed to be located in the *trans*-golgi network of melanocytes and is involved in melanosome maturation (Ginger *et al.* 2008;Wilson *et al.* 2013). Polymorphisms in this gene cause a golden phenotype in zebrafish and are associated with variations in skin color (Lamason *et al.* 2005) as well as OCA6 in humans (Stokowski *et al.* 2007, Morice-Picard *et al.* 2014). Additionally, mice with a targeted mutation in *SLC24A5* have depigmentation in the RPE, ciliary body, and iris pigment epithelium of the eye, but their coat color was grossly indistinguishable from wild-type mice (Vogel *et al.* 2008). Histological examination of the melanocytes of the hair and skin of these mice did reveal reduced pigment from wild-type littermates; however, the coat color was indistinguishable by visual inspection (Vogel *et al.* 2008). This model is especially relevant to the tiger-eye study, because it is a similar phenotype where *SLC24A5* mutations cause depigmentation of the iris but no overt coat color differences. Histological examination of horse melanocytes with the tiger-eye variants is warranted but not possible at this time.

Both variants identified here are predicted to be deleterious to protein function and therefore affect the role of *SLC24A5* in melanogenesis. Three-dimensional modeling of these mutations was not possible as no topological model exists for SLC24A5. However, a topological model has been established for the close relative SLC24A2, and all of the members of the *SLC24A* family are predicted to have similar topology, including two cation exchange transmembrane domains and a large loop connecting these two domains (Kinjo *et al.* 2003; Szerencsei *et al.* 2013). Thus, comparing our mutations to the defined topology for SLC24A2, the Tiger-eye 1 mutation (F91Y) localizes to the intraluminal loop just prior to the first transmembrane domain involved in cation ligand binding and transport. Across 100 vertebrates, the phenylalanine at position 91 is conserved, which taken together further supports our hypothesis that the Phe > Tyr is deleterious to protein function (Figure S3 in File S1).

The Tiger-eye 2 deletion would remove amino acids 292–360, which includes part of the large loop connecting the first and second cation exchange domains as well two transmembrane α helices. One of these helices forms a part of the second-predicted Na/Ca exchanger membrane domain. Thus, loss of an entire exon would likely be detrimental to the function of cation exchange. However, while detrimental to protein function, homozygosity for this deletion is not lethal, as we detected one homozygous horse with blue eyes. This horse was also heterozygous for the cream dilution (missense mutation in *SLC45A2*) as expected from its palomino coat color (Mariat *et al.* 2003). It may be that the Tiger-eye 2 deletion in its homozygous state or in conjunction with a single copy of the cream dilution is another cause of blue eyes in horses. Testing additional blue-eyed PRPF horses is necessary to confirm this hypothesis.

Unlike the mouse models and humans with OCA6, the retina of homozygous Tiger-eye 1 and compound heterozygous horses did not reveal decreased pigment on the RPE. In addition, OCA6 patients have an underdeveloped macula fovea (Wei *et al.* 2013). The fovea is the region of retina with the highest concentration of cones responsible for color vision and visual acuity (Kolb 2005). The analogous structure in the horse is known as the visual streak. While having a high concentration of ganglion cells in horses, it is more rod dominated (Gilger 2016). This may indicate that, in the horse, SLC24A5 plays a different role in ocular development and is restricted to pigmentation of the iris. Further, in humans, OCA6 is associated with nystagmus, photophobia, and decreased visual acuity, which were not noted in tiger-eye horses examined here. One of the tiger-eye horses examined was found to have an eyelid margin defect and a dermoid on the dorsal limbus OD. These collagen abnormalities are thought to be unrelated to the tiger-eye phenotype and may have some other underlying unknown genetic component or could have resulted from an injury. The homozygous Tiger-eye 2 horse was not examined by a veterinary ophthalmologist; therefore, it is unclear if Tiger-eye 2 in its homozygous state is associated with any ocular anomalies.

Both forms of melanin, pheomelanin (black/brown pigment) and eumelanin (red/yellow), are present in the human iris and differences in eye color can be explained by changes in the type and amount of melanin (Sturm and Larsson 2009). In human iris pigmented epithelium, people with brown eyes have a ratio of eumelanin to pheomelanin of 3.7 and people with green eyes have an almost equal ratio (0.88), demonstrating that the type of pigment present has a large effect on iris color (Prota *et al.* 1998). Further, monkeys with yellow iris pigmentation were reported to have a eumelanin/pheomelanin ratio of 2.3 compared to brown-eyed humans with a ratio of 4.7 and black-eyed rabbits with a ratio of 106 (Prota *et al.* 1998). In a single OCA6 patient with two pathogenic alleles in *SLC24A5*, decreased eumelanin content in the hair was noted when compared to her dark-haired brother, suggesting that the mutations in *SLC24A5* cause a decrease in eumelanin while pheomelanin is preserved (Wei *et al.* 2013). The normal ratio of eumelanin/pheomelanin in the iris of the horse has not been documented and it is unclear if the yellow iris pigmentation in tiger-eye horses is due to decreased amount of eumelanin, or an increased amount of pheomelanin. However, visual inspection of photographic records of base coat color in this study did not identify a higher incidence of tiger-eye among chestnut horses (horses that only produce pheomelanin in the coat); of the 32 tiger-eye horses, 10 were chestnut in coloration. Chestnut horses do not appear to have a more pronounced eye-color phenotype, all shades of yellow, amber, and bright orange were observed among this group of horses. Investigating how these *SLC24A5* variants affect melanin content remains to be determined.

### Conclusions

This study identified a locus on ECA 1 strongly associated with the tiger-eye phenotype in PRPF horses. Two mutations in the pigmentation gene *SLC24A5* explain the majority of iris color in this breed. Both mutations are predicted to be deleterious and are in highly conserved regions, supporting the conclusion that these two mutations are causative. In contrast to the pleiotropic effects of SLC24A5 mutations in humans, the Tiger-eye 1 variant identified in the current research only appears to affect iris color. Only one Tiger-eye 2 homozygote was identified, and it was not examined by an ophthalmologist, so further research is needed on the effects of this variant in its homozygous state. Future research investigating the genetics of iris color variation in vertebrates should include *SLC24A5* for consideration as a candidate gene based on the novel role we present here in horses.

## Supplementary Material

Supplemental material is available online at www.g3journal.org/lookup/suppl/doi:10.1534/g3.117.043786/-/DC1.
