# Supplementary material for: Two Variants in SLC24A5 Are Associated with “Tiger-Eye” Iris Pigmentation in Puerto Rican Paso Fino Horses
Source: G3 (Bethesda). 2017 Jun 27;7(8):2799–806. doi: 10.1534/g3.117.043786 (PMC5555483; doi:10.1534/g3.117.043786)
Supplement: Supplementary file 1 [file 2799FileS1.docx]

**Supplemental Tables and Figures**

**Table S1 Primers and enzymes for allele specific PCR, PCR-RFLP, and sequencing *SLC24A5***.

| **Primer Name** | **Forward primer** | **Reverse primer** | **Enzyme** |
| --- | --- | --- | --- |
| Exon 7 Deletion | ATTGGGAAGTGGAGAGCACT | GATCCCTTGACTTTACCTCTGG | N/A |
| Exon 7 Wild Type | TCCTGGTTTGGATGGTCACA | GATCCCTTGACTTTACCTCTGG | N/A |
| Exon 2 Mutant | CAATCGAGATGGACAGAATTGTT | GTTTCTATTGTCTGTGACGAGTAGTA | N/A |
| Exon 2 Wild Type | CAATCGAGATGGACAGAATTGTT | GTTTCTTTCTATTGTCTGTGACGAGTCCTT | N/A |
| BIEC2_61330 | CTTGGCAGTCTCTCCTTTGG | CAGTCAAGATGGTGGCGTG | MspI |
| UKUL310 | TCCTTGAGAAGAAATGGCTCA | TTCACTTCAATGGCTGGAAA | MaeIII |
| BIEC2_60719 | AAGGATACGGAAAATGAAGACG | AGAACCACGAACAGCAACTG | HpyCH4V |
| BIEC2_61972 | TTTTTATGTTCCTAATTTTACAACCTG | GAAATCGTGTACCAACAGGTCA | MspI |
| **Sanger Sequencing Primers** |  |  |  |
| SLC24A5-Ex1 | GGTTCAAGCCACCATCATTT | AGGCCTGCACAGAATCATCT |  |
| SLC24A5-Ex2 | CATCAGAGATCTAGCTTCTCCTTG | CCAGGCAGTGCAGTAGACAA |  |
| SLC24A5-Ex3-4 | ACAAGCCCTACAGCTGGAAA | GACCAAGAGCTATTTGTGGGTTA |  |
| SLC24A5-Ex5 | AATCTCCTTGGCATCTGTGC | TCTTAGAGGAGGGCGGCTAC |  |
| SLC24A5-Ex6 | TTGCCTTGATTGTTCTATTGCT | GCTTCCAAACCTGCAAAGAC |  |
| SLC24A5-Ex7 | GAAGCCAGATTACGCCCATA | TCAGCTCTGCCCATGTAGTG |  |
| SLC24A5-Ex7-2 | ATCCTGGTTTGGATGGTCAC | TCAGCTCTGCCCATGTAGTG |  |
| SLC24A5-In6 | TTTGAGCAGCAGCAAAACAC | TTTCTTGATTTGGGGCCTTA |  |
| SLC24A5-Ex8 | GGATTCTAAAGCAGGTTTCCA | TCCTTCCTCTTGAGTCACTCTTT |  |
| SLC24A5-Ex9 | CATCTGAAATGTGCATTAAATCG | TCCTTGAGAAGAAATGGCTCA |  |
| SLC24A5-In2+1 | GCACTGCCTGGCTGTTAGTA | ACTGGCATTTCAGCAGAGGT |  |
| SLC24A5-ln2+2 | GTTACCCAGGAGAAATTTACCA | TCCCACCTAGGGATAAAAAGG |  |
| SLC24A5-ln2+3 | ATGTGGGATTGATGGATGC | TAAATTTCCGGTGTCCTTGG |  |
| SLC24A5-ln2+4 | GCCACCATAACATGGTTGTTT | GAAGGGAGTACAGCAACAGCA |  |
| SLC24A5-ln2+5 | CAGGGCATTTTACCTTTGGA | CAGATGTGCCCTAGCTTTGC |  |
| SLC24A5-ln2+6 | AGGAATAGCTCTGGGGAAGG | AGGGCATAAACCCTGAGTCC |  |
| SLC24A5-ln2+7 | TGAGGTGTCTCTAGTCTGGGAGT | CGATTTAAAACGTGGAACCATT |  |

**Table S2 Phenotypes and Available Genotypes of the Horses Included in the Pedigree Analysis (N=41) Organized By Mating Types**

| **Tiger x Tiger = Tiger Progeny (N=5)** | | | |
| --- | --- | --- | --- |
| **Progeny ID** | **Phenotype : Genotype** | **Sire Phenotype : Genotype** | **Dam Phenotype : Genotype** |
| 09-57T | T : Tiger-eye 1/Tiger-eye 2 | T : No DNA Available | T : No DNA Available |
| 09-68T | T : Tiger-eye 1/Tiger-eye 2 | T : No DNA Available | T : No DNA Available |
| 09-99T | T : Tiger-eye 1/Tiger-eye 1 | T : Tiger-eye 1/Tiger-eye 1 | T : No DNA Available |
| X80T | T : No DNA Available | T : No DNA Available | T : No DNA Available |
| 16-212T | T : Tiger-eye 1/Tiger-eye 1 | T : Tiger-eye 1/Tiger-eye 1 | T : Tiger-eye 1/+* |
| **Tiger x Brown = Tiger Progeny (N=6)** | | | |
| **Progeny ID** | **Phenotype : Genotype** | **Sire Phenotype : Genotype** | **Dam Phenotype : Genotype** |
| 09-01T | T : Tiger-eye 1/Tiger-eye 1 | T : No DNA Available | B : No DNA Available |
| 09-35T | T : Tiger-eye 1/Tiger-eye 1 | B : No DNA Available | T : No DNA Available |
| 09-74T | T : Tiger-eye 1/Tiger-eye 1 | T : Tiger-eye 1/Tiger-eye 1 | B : Tiger-eye 1/+ |
| 09-76T | T : Tiger-eye 1/Tiger-eye 1 | T : No DNA Available | B : Tiger-eye 1/+ |
| X135T | T : No DNA Available | T : No DNA Available | B : No DNA Available |
| X21T | T : No DNA Available | B : No DNA Available | T : No DNA Available |
| **Brown x Brown = Tiger Progeny (N=1)** | | | |
| **Progeny ID** | **Phenotype : Genotype** | **Sire Phenotype : Genotype** | **Dam Phenotype : Genotype** |
| 12-223T | T : Tiger-eye 1/Tiger-eye 1 | B : Tiger-eye 1/+ | B : Tiger-eye 1/+ |
| **Brown x Brown = Brown Progeny (N=11)** | | | |
| **Progeny ID** | **Phenotype : Genotype** | **Sire Phenotype : Genotype** | **Dam Phenotype : Genotype** |
| 09-23B | B : Tiger-eye 1/+ | B : +/+ | B : No DNA Available |
| 09-80B | B : +/+ | B : +/+ | B : No DNA Available |
| 09-81B | B : Tiger-eye 1/+ | B : +/+ | B : No DNA Available |
| 09-72B | B : Tiger-eye 1/+ | B : +/+ | B : No DNA Available |
| 09-95B | B : Tiger-eye 1/+ | B : +/+ | B : No DNA Available |
| 11-141B | B : +/+ | B : +/+ | B : +/+ |
| 11-133B | B : Tiger-eye 1/+ | B : +/+ | B : Tiger-eye 1/+ |
| 09-02B | B : Tiger-eye 1/+ | B : Tiger-eye 1/+ | B : No DNA Available |
| 09-58B | B : +/+ | B : No DNA Available | B : No DNA Available |
| 11-188B | B : Tiger-eye 1/+ | B : +/+ | B : Tiger-eye 1/+ |
| 11-144B | B : +/+ | B : No DNA Available | B : +/+ |
| **Brown x Tiger = Brown Progeny (N=18)** | | | |
| **Progeny ID** | **Phenotype : Genotype** | **Sire Phenotype : Genotype** | **Dam Phenotype : Genotype** |
| 09-31B | B : Tiger-eye 1/+ | T : Tiger-eye 1/Tiger-eye 1 | B : +/+ |
| 09-34B | B : Tiger-eye 1/+ | T : Tiger-eye 1/Tiger-eye 1 | B : No DNA Available |
| 09-03B | B : Tiger-eye 1/+ | T : Tiger-eye 1/Tiger-eye 1 | B : No DNA Available |
| 09-05B | B : Tiger-eye 1/+ | T : Tiger-eye 1/Tiger-eye 1 | B : No DNA Available |
| 09-06B | B : Tiger-eye 1/+ | T : Tiger-eye 1/Tiger-eye 1 | B : No DNA Available |
| 09-89B | B : Tiger-eye 1/+ | T : Tiger-eye 1/Tiger-eye 1 | B : No DNA Available |
| X75B | B : No DNA Available | B : +/+ | T : No DNA Available |
| 09-38B | B : Tiger-eye 1/+ | B : +/+ | T : No DNA Available |
| 09-42B | B : Tiger-eye 1/+ | B : +/+ | T : Tiger-eye 1/ Tiger-Eye 2 |
| 12-226B | B : Tiger-eye 2/+ | B : +/+ | T : Tiger-eye 1/ Tiger-Eye 2 |
| 09-15B | B : Tiger-eye 1/+ | B : Tiger-eye 1/+ | T : Tiger-eye 1/Tiger-eye 1 |
| 09-90B | B : Tiger-eye 1/+ | B : Tiger-eye 1/+ | T : Tiger-eye 1/Tiger-eye 1 |
| 12-219B | B : Tiger-eye 2/+ | B : Tiger-eye 1/+ | T : Tiger-eye 1/Tiger-eye 2 |
| 11-121B | B : Tiger-eye 1/+ | B : No DNA Available | T : No DNA Available |
| 09-84B | B : Tiger-eye 1/+ | B : No DNA Available | T : Tiger-eye 1/Tiger-eye 2 |
| 09-77B | B : Tiger-eye 2/+ | B : No DNA Available | T : Tiger-eye 1/Tiger-eye 2 |
| 11-178B | B : Tiger-eye 1/+ | B : No DNA Available | T : No DNA Available |
| X8B | B : No DNA Available | B : +/+ | T : No DNA Available |

*****This is one of the four outliers (Tiger-eye 1/+ ) noted in the discussion and shown in Figure S2D.

**Table S3 Coat Color of Tiger-eye horses of different shades**

|  |  | **Coat Color** |  |
| --- | --- | --- | --- |
|  | Bay | Chestnut | Black |
| Yellow - Tiger-eye | 7 | 3 | 0 |
| Amber - Tiger-eye | 10 | 4 | 1 |
| Orange - Tiger-eye | 4 | 3 | 0 |
| All Tiger-eye | 21 | 10 | 1 |

**Table S4 The Ten Most Strongly Associated SNPs from Chi-squared Test Using a Recessive Model (n=24)**

| Marker | Location | Chi-Squared P | Minor Allele | Major Allele | Minor Allele Frequency | Major Allele Frequency |
| --- | --- | --- | --- | --- | --- | --- |
| BIEC2_60719 | chr1:138425099 | 6.76E-06 | A | G | 0.27 | 0.73 |
| BIEC2_61972 | chr1:141804174 | 7.12E-06 | G | A | 0.23 | 0.77 |
| BIEC2_61330 | chr1:139309629 | 3.47E-05 | A | G | 0.33 | 0.67 |
| BIEC2_59902 | chr1:136798782 | 4.15E-05 | A | G | 0.19 | 0.81 |
| BIEC2_62072 | chr1:141978176 | 4.15E-05 | G | A | 0.21 | 0.79 |
| BIEC2_844267 | chr4:3456307 | 4.93E-05 | G | A | 0.375 | 0.625 |
| UKUL310 | chr1:141657822 | 5.91E-05 | A | G | 0.3 | 0.7 |
| BIEC2_61415 | chr1:139675202 | 6.52E-05 | A | G | 0.2 | 0.8 |
| BIEC2_58699 | chr1:134805747 | 1.40E-04 | G | A | 0.375 | 0.625 |
| BIEC2_62199 | chr1:134986606 | 1.40E-04 | C | A | 0.375 | 0.625 |

**Table S5 Shade of Tiger-Eye and Genotype of the 32 Tiger-eye Horses**

|  |  | **Genotype** |  |
| --- | --- | --- | --- |
|  | Tiger-eye 1/ Tiger-eye 1 | Tiger-eye 1/ Tiger-eye 2 | Tiger-eye 1/+ |
| Yellow | 6 | 3 | 1 |
| Amber | 11 | 4 | 0 |
| Orange | 3 | 1 | 3 |
| All Tiger-eye | 20 | 8 | 4 |


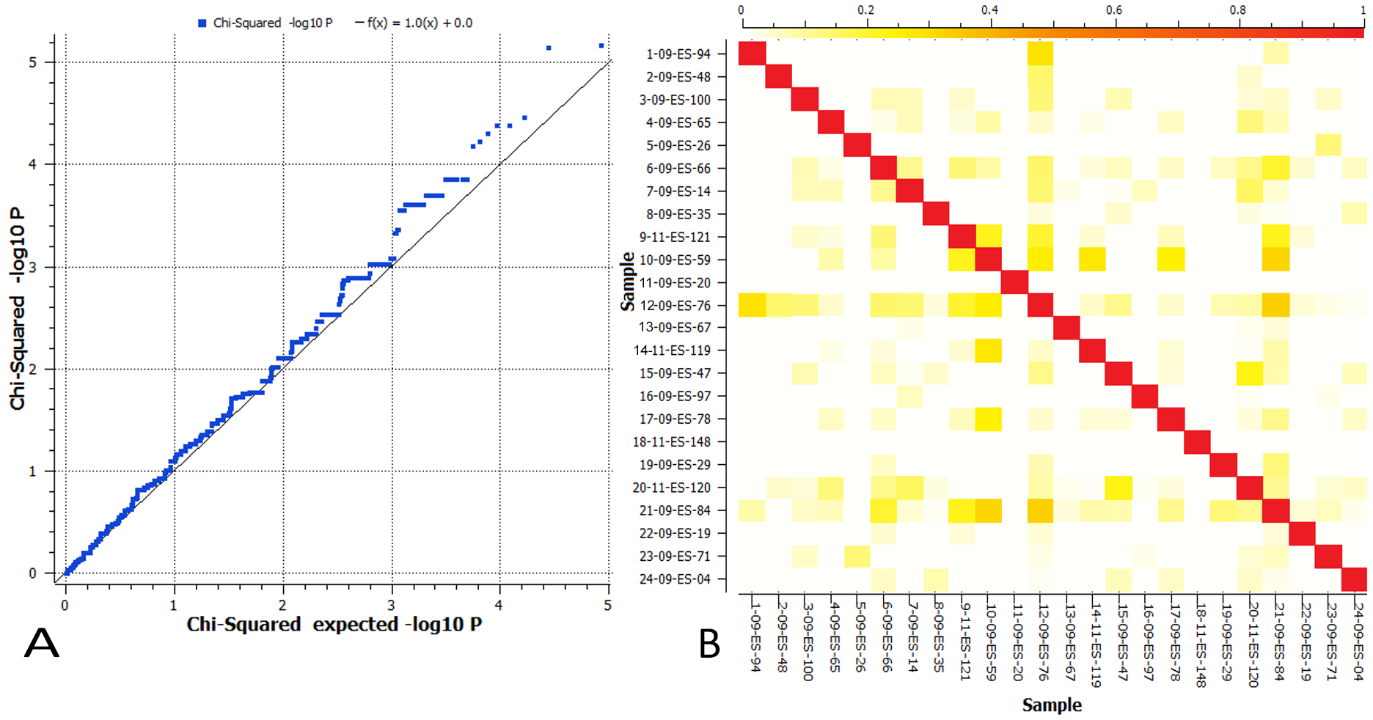


**Figure S1 Population Structure** A) Q-Q plot of chi-squared – log10 P versus chi-squared expected –log10p based on basic allelic association testing. Deviation from this line is represents the genomic inflation in the population (λ= 1.21). B) Identity by descent (IBD) heat map, demonstrating the relatedness between individuals in our population set.


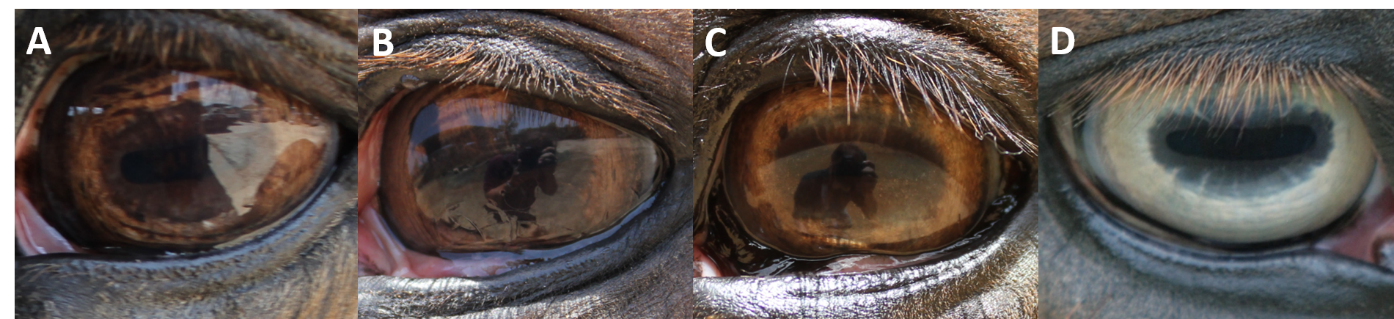


**Figure S2 Eye color of horses not explained by Tiger-eye 1 or Tiger-eye 2 alleles.** Eye colors of the four-outlier horses that were classified as tiger-eye, but were not homozygous for the exon 2 variant or were not compound heterozygotes. All four horses were heterozygous for the Tiger-eye 1 allele. Horses A, B, C were categorized as Orange-Tiger, which is the darker end of the spectrum for tiger-eye. Horse D was categorized as the lightest shade Yellow-Tiger.


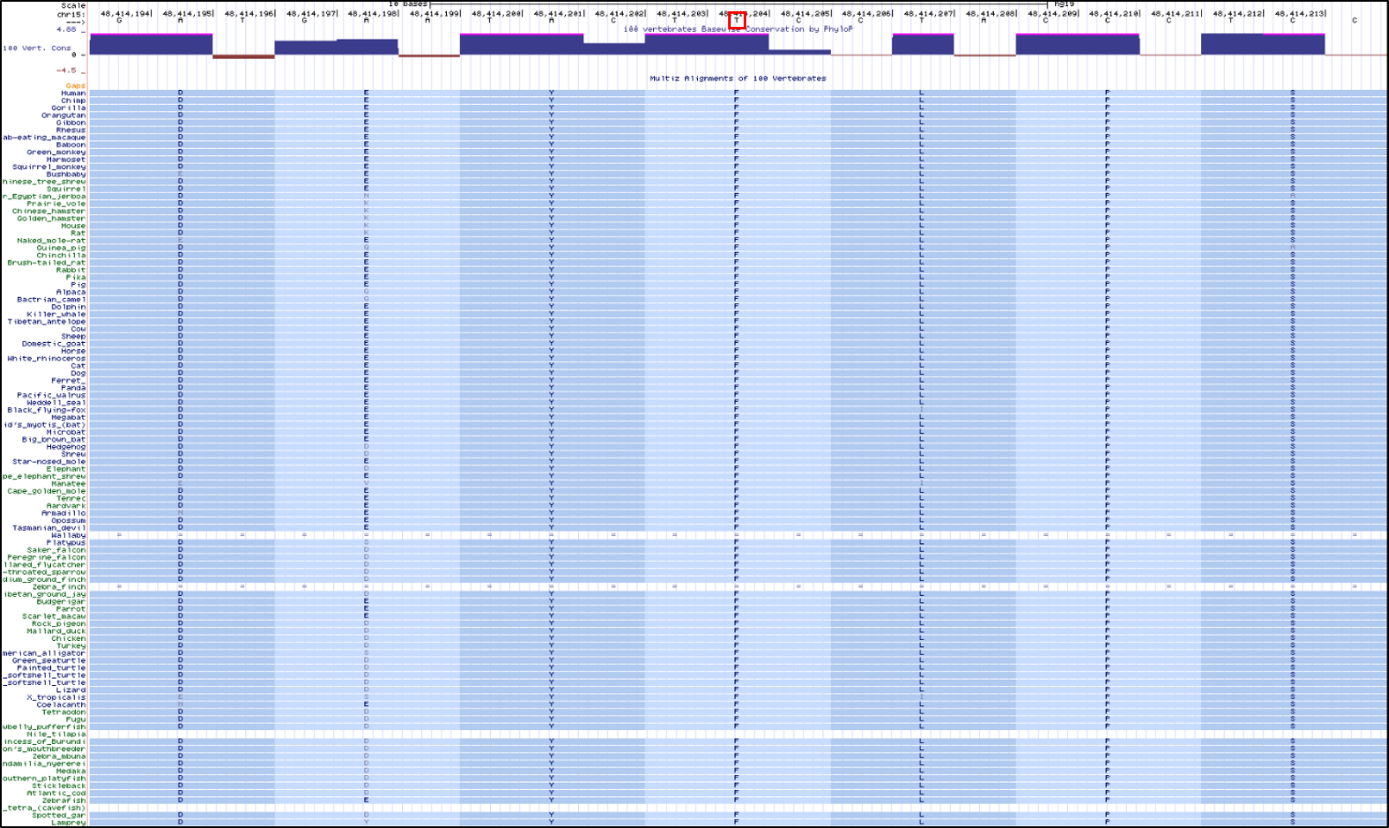


**Figure S3 Multispecies Alignment of Phe.91 Demonstrating the Conservation Across Vertebrates.** The location of the Tiger-eye 1 variant is boxed in red. Nucleic acid and protein sequence conservation across 100 vertebrates is represented for the Phe.91allele and three flanking codons (upstream and downstream). Image was prepared using the human reference genome (hg38) and Vertebrate Multiz Alignment & Conservation (100 Species) track **(**[**www.uscsgenomebrowswer**](http://www.uscsgenomebrowswer)**.org).**
